# Antibody Protection against Long-Term Memory Loss Induced by Monomeric C-Reactive Protein in a Mouse Model of Dementia

**DOI:** 10.3390/biomedicines9070828

**Published:** 2021-07-16

**Authors:** Elisa García-Lara, Samuel Aguirre, Núria Clotet, Xenia Sawkulycz, Clara Bartra, Lidia Almenara-Fuentes, Cristina Suñol, Rubén Corpas, Peter Olah, Florin Tripon, Andrei Crauciuc, Mark Slevin, Coral Sanfeliu

**Affiliations:** 1 Institut d’Investigacions Biomèdiques de Barcelona (IIBB), CSIC and IDIBAPS, 08036 Barcelona, Spain; elisaglara21@gmail.com (E.G.-L.); samuelaguirreinfantes@gmail.com (S.A.); nuria.cg07@gmail.com (N.C.); clara.bartra@iibb.csic.es (C.B.); almenara.lidia.22@gmail.com (L.A.-F.); cristina.sunol@iibb.csic.es (C.S.); rubencorpas@gmail.com (R.C.); 2 School of Life Sciences, John Dalton Building, Manchester Metropolitan University, Manchester M15 6BH, UK; XENIA.SAWKULYCZ@stu.mmu.ac.uk; 3 Genetics Department, George Emil Palade University of Medicine, Pharmacy, Science and Technology of Targu Mures, 540142 Targu Mures, Romania; olah_peter@yahoo.com (P.O.); tripon.florin.2010@gmail.com (F.T.); andrei.crauciuc@gmail.com (A.C.)

**Keywords:** monomeric C-reactive protein (mCRP), biomarker, Alzheimer’s disease, mouse model of mCRP dementia

## Abstract

Monomeric C-reactive protein (mCRP), the activated isoform of CRP, induces tissue damage in a range of inflammatory pathologies. Its detection in infarcted human brain tissue and its experimentally proven ability to promote dementia with Alzheimer’s disease (AD) traits at 4 weeks after intrahippocampal injection in mice have suggested that it may contribute to the development of AD after cerebrovascular injury. Here, we showed that a single hippocampal administration of mCRP in mice induced memory loss, lasting at least 6 months, along with neurodegenerative changes detected by increased levels of hyperphosphorylated tau protein and a decrease of the neuroplasticity marker *Egr1*. Furthermore, co-treatment with the monoclonal antibody 8C10 specific for mCRP showed that long-term memory loss and tau pathology were entirely avoided by early blockade of mCRP. Notably, 8C10 mitigated *Egr1* decrease in the mouse hippocampus. 8C10 also protected against mCRP-induced inflammatory pathways in a microglial cell line, as shown by the prevention of increased generation of nitric oxide. Additional in vivo and in vitro neuroprotective testing with the anti-inflammatory agent TPPU, an inhibitor of the soluble epoxide hydrolase enzyme, confirmed the predominant involvement of neuroinflammatory processes in the dementia induced by mCRP. Therefore, locally deposited mCRP in the infarcted brain may be a novel biomarker for AD prognosis, and its antibody blockade opens up therapeutic opportunities for reducing post-stroke AD risk.

## 1. Introduction

Sustained neuroinflammation is a risk factor for age-related diseases, including cerebrovascular injuries and Alzheimer’s disease (AD) [1,2]. Systemic inflammatory conditions may trigger or aggravate a range of cardiovascular and metabolic diseases that also contribute to brain dysfunction and dementia [3,4]. Indeed, inflammatory processes are increasingly being considered the culprits of frailty and disease in the elderly. As a result, the search for reliable inflammatory biomarkers and intervention targets to combat dementia and other disabling conditions has intensified.

C-reactive protein (CRP) is a widely used peripheral marker of inflammatory processes. It was identified by Tillet and Francis in 1930 in the blood of patients with pneumococcal infection [5], and its synthesis in the liver is stimulated by circulating pro-inflammatory cytokines during the acute phase of infection [6]. Chronic moderately elevated expression of CRP is associated with an increased risk of a wide range of diseases [7,8,9], and CRP has been proposed as a biomarker for several inflammatory-associated ailments [10,11]. High-sensitivity CRP (hsCRP) testing, able to detect values under 3 mg/L, is a sensitive test of low-grade inflammation, whereby the normal population presents mean hsCRP levels of 2 mg/L, but desirable values are less than 1 mg/L [8]. CRP molecules that circulate in blood have the structure of a pentamer with five identical monomers in a β-jelly roll characteristic of the pentraxin superfamily of proteins. Pentraxins are evolutionary conserved proteins involved in immunological responses. Interestingly, native pentameric CRP may suffer conformational changes into the isoforms, termed pCRP*, or dissociate into modified or monomeric CRP (mCRP), where pCRP* and mCRP expose functionally active neoepitopes that carry out highly pro-inflammatory functions [12]. Molecular studies ex vivo have shown that CRP activation occurs in damaged vessels and other tissues, where pro-inflammatory forms may activate immune cells and complement reactions [13]. Deposition of mCRP, which has a much lower aqueous solubility than CRP, has been shown in the brain in infarcted areas of AD patients [14] and in regions with amyloid burden [15], in atherosclerotic plaques in vascular disease [16] and in other foci of inflammatory tissue injuries [17,18]. An unbound fraction of mCRP has also recently been reported in serum of patients with high levels of circulating native CRP (>100 mg/L, in the range of acute inflammatory response), although the dissociation site of origin is unclear [19]. Furthermore, mCRP has been detected in U937-derived macrophages [20] and exosomes derived from monocytes of patients with coronary artery disease [21]. Additionally, other cell types such as neurons in the AD brain may produce CRP, albeit in lower amounts than hepatocytes [22]. New specific antibodies and upcoming analytical tools for quantifying the different forms of CRP will help to understand the functionality of the complex CRP system [23]. However, whereas CRP is essential in host defense and clearance of apoptotic cells, mCRP seems to exert severe pro-inflammatory and tissue damaging effects and is emerging as a target for anti-inflammatory therapies in chronic diseases [24,25].

Specific targeting of mCRP can be a therapeutic approach in areas in which rapid increases in its local generation are expected, such as stroke-affected brain areas, in order to halt subsequent neurodegeneration and dementia. The prevalence of dementia in stroke survivors is about 30%, and a high proportion of these patients suffer AD (in addition to those with either vascular or mixed AD plus vascular dementia) [26]. Furthermore, cerebrovascular pathological findings are common in post-mortem AD brain [27]. Inflammatory damage spreading from small blood vessels and linked dysregulation of amyloid β metabolism in the neurons have been implicated in the origin of AD [28]. It is known that mCRP accumulates in brain micro-vessels after ischemic stroke [14], where it promotes aberrant angiogenesis [29], accumulation of amyloid β [30] and probably de novo synthesis of amyloid β [31]. Therefore, mCRP may cause both vascular and neuronal degeneration and underlie the processes leading to poststroke dementia [14,28]. Furthermore, in a previous experimental study, we demonstrated that mCRP injected into the hippocampus of mice induces memory loss after 4 weeks, as well as other traits of AD such as mild amyloid and tau pathology in neurons of the hippocampus and cortical areas [30]. We have also shown that intrahippocampal mCRP may partially spread through the microvasculature to the cortical and hypothalamic areas [32]. Neurotransmission dysfunction in cortical-limbic areas causes behavioral alterations, known as ‘behavioral and psychological symptoms of dementia’ (BPSDs), that are common in patients of AD and other dementia [33]. Anxiety, depression and apathy are some of these behaviors that may be reproduced in AD mouse models [34].

We hypothesize that mCRP is a biomarker of AD prognosis in the infarcted brain and probably also in other inflammatory brain pathologies that increase the risk of AD. In this study, we wished to further characterize the mCRP mouse model of poststroke dementia to investigate whether memory loss, the concomitant presence of BPSDs and pathological pro-neurodegenerative processes are maintained in the long term, up to 6 months after intrahippocampal injection of mCRP. We also used a specific antibody against mCRP, the monoclonal 8C10 antibody [29], to test whether blockade of mCRP may halt subsequent damaging mechanisms and confer long-term neuroprotection in this proposed model of neurodegeneration. We aimed to prove that mCRP can be therapeutically inhibited once injected into the mouse hippocampus. We also aimed to analyze the inflammatory mechanisms in mCRP-induced dementia by using 1-trifluoromethoxyphenyl-3-(1-propionylpiperidin-4-yl)-urea (TPPU), an inhibitor of the epoxide hydrolase enzyme, since inhibition of this enzyme has recently been proposed as a protective mechanism against neuroinflammation [35]. Finally, we wished to characterize the modulation of the well-known inflammatory pathways of nitric oxide by mCRP, 8C10 and TPPU in an in vitro setting using the BV2 microglial cell line.

## 2. Material and Methods

### 2.1. Experimental Agents

Monomeric C-reactive protein (mCRP) was generated from a commercial source of recombinant human native CRP using the Potempa method [32]. Briefly, 1 mL commercial CRP protein was chelated in a 1:1 ratio with EDTA/urea buffer (10 mM EDTA, 8M urea) and incubated at 37 °C for 2 h and further dialyzed (20 kDa MWCO) in buffer (25 mM Tris–HCl, 50 mM NaCl; pH 8.3) for 24 h to recover a solution of pure monomers of mCRP. Native CRP was obtained from YO Proteins (Ronninge, Sweden); all other reagents were from Sigma (St. Louis, MO, USA) where not otherwise indicated. 

Mouse monoclonal antibody against human mCRP clone 8C10 was obtained from Dr L.A. Potempa by hybridoma technology and fully characterized, as described previously [29,36]. The non-purified hybridoma culture supernatant was directly used as an experimental 8C10 solution. We have shown its ability to block mCRP, preventing the activation of U937 monocytes [13].

The soluble epoxide hydrolase inhibitor TPPU was used as a reference anti-inflammatory agent. TPPU was purchased from MedChemExpress (Monmouth Junction, NJ, USA).

### 2.2. Animals and Experimental Design

One hundred and fifty-two C57BL/6J male mice were used in this study. Mice bred by Janvier Labs (France) were purchased from Novaintermed (Pipera, Romania) and maintained for the study in the Animal Unit of the University of Barcelona (UB), Spain. Animals were individually housed in Makrolon cages (Techniplast, Buguggiatte, Italy) with free access to food and water in a temperature-controlled room (22 ± 2 °C) with a 12 h light/12 h dark cycle. All the animal procedures, including surgery, behavioral testing and necropsies, were performed at the UB animal facilities. The study design and protocols were approved by the UB Ethics Committee for Animal Experimentation (Comitè Ètic d’Experimentació Animal, CEEA-UB) under the guidelines of the Animal Experimentation Commission of the Autonomous Government of Catalonia (Comissió d’Experimentació Animal, Generalitat de Catalunya) (Approval references: #6991 and #10921). All procedures were carried out in accordance with the Directive 214/97 of the Generalitat de Catalunya, Spanish legislation (Real Decreto 1386/2018), and the European Union (EU) Directive 2010/63/EU for animal experiments.

**Figure 1:**
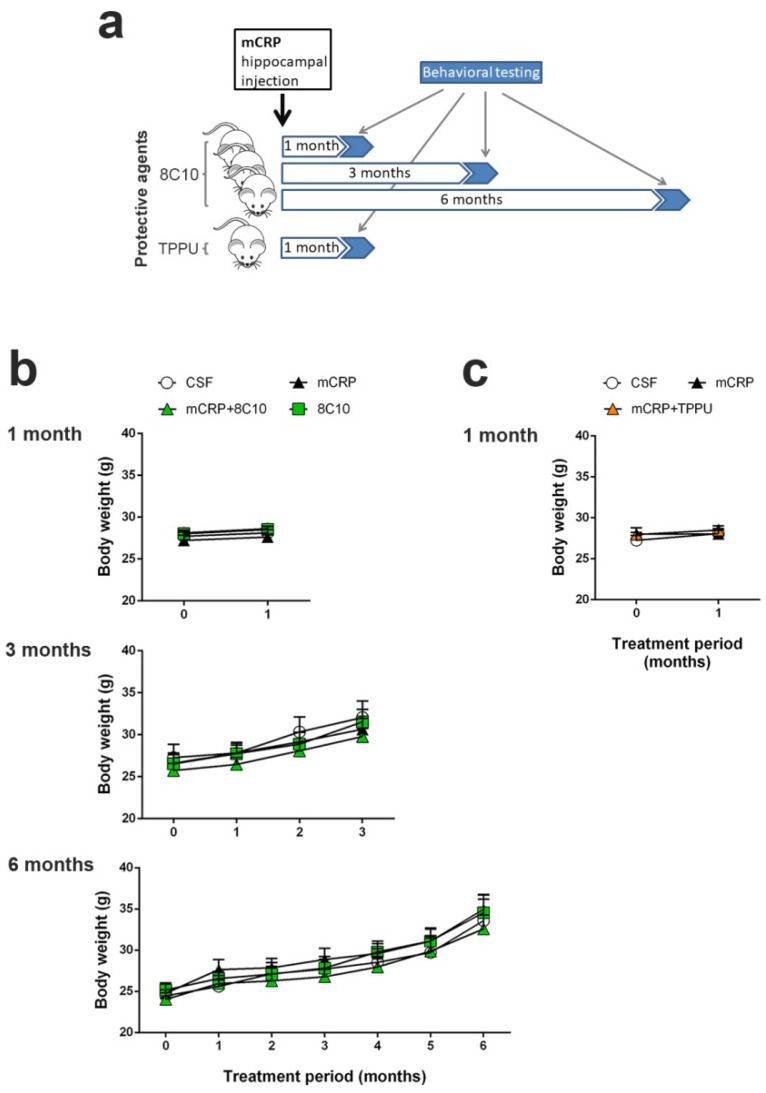
Monomeric C-reactive protein (mCRP) treatment in C57BL/6J male mice. (**a**) Scheme of the experimental design (see Methods Section for details). (**b**) Body weight control for the 1-, 3- and 6-month studies assaying a protective treatment with the anti-mCRP antibody 8C10 and (**c**) for the 1-month study assaying a protective treatment with Trifluoromethoxyphenyl-3-(1-propionylpiperidin-4-yl)-urea (TPPU). Progression of body weight confirmed the absence of unwanted systemic effects. Values are mean ± SEM ((**b**) 1-month graph: CSF N = 6, mCRP N = 9, mCRP + 8C10 N = 12 and 8C10 N = 6; 3-month graph: CSF N = 13, mCRP N = 12, mCRP + 8C10 N = 12 and 8C10 N = 11; 6-month graph; CSF N = 11, mCRP N = 11, mCRP + 8C10 N = 12, 8C10 N = 10. (**c**) CSF N = 9, mCRP N = 9 and mCRP + TPP N = 9).

We performed 3 independent studies with intrahippocampal mCRP treatment over periods of 1 month, 3 months and 6 months respectively, to test neuroprotection by 8C10. Animals received a single bilateral intrahippocampal injection (see Section 2.3 for details) of 1 µL mCRP at 3.5 µg/µL and/or 1 µL 8C10 antibody solution. Dosage was selected in a preliminary study. Volume in the mice dosed with either solution was completed to 2 µL, with 1 µL of artificial solution of CSF (NaCl 148 mM, KCl 3 mM, CaCl_2_ 1 mM, MgCl_2_ 0.8 mM, Na_2_HPO_4_ 0.8 mM, NaH_2_PO_4_ 0.2 mM). Mice in the control group received a bilateral injection of 2 µL CSF. Therefore, for each study, the experimental groups were as follows: control (CSF), mCRP, mCRP plus anti-mCRP antibody (mCRP + 8C10) and anti-mCRP antibody (8C10). Experimental procedures were established in a previous 1-month study with the mCRP mouse model of dementia [30]. An additional study was performed with the same intrahippocampal mCRP treatment for 1 month to test neuroprotection by oral TPPU dosing. TPPU was administered orally beginning 2 days before a single bilateral mCRP or CSF injection, and throughout the study in the drinking water, mixed with cyclodextrin 3% to improve its solubility. The control group received the same dose of cyclodextrin in the drinking water. TPPU was added to the drinking water at a concentration that yielded a daily dose of 5 mg/kg body weight. The initial concentration of TPPU was established in a preliminary study of water consumption per mouse. Thereafter, the water consumption and body weight of the mice were measured twice a week and the TPPU concentration was adjusted accordingly in a freshly prepared drinking solution. This treatment regimen was found to be neuroprotective in AD mouse models [37]. The experimental groups were: control (CSF), mCRP and mCRP plus TPPU (mCRP + TPPU). A schematic drawing of the experimental design is shown in Figure 1a. The number of animals per group was as follows: (i) 1-month mCRP/8C10 study, CSF N = 6, mCRP N = 9, mCRP + 8C10 N = 12 and 8C10 N = 6; (ii) 3-month mCRP/8C10 study, CSF N = 13, mCRP N = 12, mCRP + 8C10 N = 12 and 8C10 N = 11; (iii) 6-month mCRP/8C10 study, CSF N = 11, mCRP N = 11, mCRP + 8C10 N = 12 and 8C10 N = 10; (iv) 1-month mCRP/TPPU study, CSF N = 9, mCRP N = 9 and mCRP + TPPU N = 9. Mice were visually inspected and weighed on a regular basis throughout the studies in order to control their general health status. All animals survived to termination.

Heterozygous transgenic AD mice of the strain 5XFAD [38] and their wild-type siblings (WT), 7-month-old males, were used for a selected analysis (see Section 2.6). All mice, N = 8 per group, were bred from first progenitors obtained from Jackson Laboratory (Bar Harbor, ME, United States) and maintained in the same housing conditions as described for the C57BL/6J mice.

### 2.3. Hippocampal Surgery and Treatment Administration

Treatments of mCRP and 8C10 were administered in the CA1 region of the mouse hippocampus by stereotactic surgery procedures, as previously performed [30]. Three-month-old C57BL/6J mice were anesthetized with 100 mg/kg ketamine (Ketolar 50 mg/mL, Pfizer, Alcobendas, Madrid, Spain) and 10 mg/kg xylazine (Rompun 2%, Bayer, Leverkusen, Germany) mixture i.p., and immobilized in a stereotactic apparatus (David Kopf Instruments, Tujunga, CA, USA). The experimental agent solutions were infused bilaterally into the CA1 area of the hippocampus. Injections were performed at a rate of 5 × 10^−4^ mL/min at coordinates relative to Bregma of −2 mm A/P, ±1.3 mm M/L, −1.6 mm V/D. Two microliters of each solution were delivered to the application point with a 2 µL 25-gauge 7000 series Neuros Syringe (Hamilton Central Europe S.R.L., Giarmata, Romania). The syringe was attached to a micro-infusion pump (Bioanalytical systems Inc., West Lafayette, IN, USA) and left in position for 5 min after delivery in order to prevent the solution from surging back.

### 2.4. Behavioral Testing

All animals were tested for behavioral changes induced by the specific treatments. Age at testing was 4, 6 and 9 months for the 1-, 3- and 6-month mCRP exposure studies, respectively. A battery of tests was applied in daily consecutive sessions, as was performed in previous studies [39,40]. After a handling habituation, animals were analyzed for sensorimotor changes and BPSD-like behaviors, such as neophobia, apathy to exploration, anxiety and depression. Learning and memory were analyzed with tests focused on recognition memory and spatial memory.

Handling habituation was performed to minimize animal stress during subsequent testing. In the handling procedure, the mouse was picked up by its tail and held in the hand for 2 min, and mice were allowed to move along the experimenter’s arm before being returned to their home cage. This procedure was repeated twice a day for 3 days with all mice. Gloves and cover sleeves were cleaned with 70° ethanol to eliminate olfactory cues between the mice.

The following specific tests were applied to all mice except for the two alternative spatial memory tests, as indicated.

Sensorimotor responses. Visual reflex and posterior leg extension reflex were measured by holding the animal by its tail and slowly lowering it toward a black surface. Motor coordination and equilibrium were assessed with a 20 s trial by the distance covered and the latency to fall off a horizontal wooden rod and a metal wire rod. Each trial was performed twice. Prehensility and motor coordination were measured as the distance covered on the wire hang test, in which the animal was allowed to cling from the middle of a horizontal wire (2 mm diameter × 40 cm length) with its forepaws for two trials of 5 s and a third trial of 60 s.

Open-field test. This test analyzes general behavior and activity. Mice were placed in the center of the apparatus (home-made, wooden, white, 55 × 55 cm surface and 25 cm high walls) and observed for 5 min. Patterns of horizontal locomotor activity (distance covered and thigmotaxis) and vertical movement (rearings) were analyzed throughout the test. Initial freezing, self-grooming behavior and the number of urine spots and defecation boli were also recorded. A computerized tracking system (SMART v3.0, Panlab S.A., Barcelona, Spain) was used to measure the distance covered, along with the ambulatory pattern.

Corner test. Neophobia to a new home-cage was assessed by introducing the animal into the center of a standard square cage (Makrolon cage 35 × 35 × 25 cm) with fresh bedding, and counting the number of corners visited and rearings during a period of 30 s. The latency of the first rearing was also recorded.

Boissier’s four-hole-board test. Exploratory and apathy-like behavior were measured as the number of head dips and time spent head-dipping on each of the four holes (3 cm diameter) equally spaced in the floor of the hole-board (woodwork white box of 32 × 32 × 32 cm). The latencies of movement, first dipping and four holes dipping were also recorded.

Dark and light box test. Anxiety-like behavior was measured in a dark–light box. The apparatus consisted of two compartments (black: 27 × 18 × 27 cm with a red light; white: 27 × 27 × 27 cm with white lighting intensity of 600 lux) connected by an opening (7 × 7 cm). The mice were introduced into the black compartment and observed for 5 min. The latency to enter the lit compartment, the time spent in the lit compartment, the number of crossings to each compartment and the vertical movement (number of rearings) were recorded.

Tail suspension test. To assess depression-like behavior, mice were suspended by the tail 30 cm above the surface. The tail was fixed with adhesive tape at 1 cm from its tip. The duration of immobility (defined as the absence of all movements except for those required for respiration) was scored over a 6 min period.

Novel object recognition test (NORT). This test assesses recognition memory. It is based on the spontaneous tendency of rodents to spend more time exploring a novel object than a familiar one. Animals were placed in the middle of a black rectangular box (30 × 40 × 30.5 cm) with a lighting intensity of 100 lux. The objects to be discriminated were made of plastic (6–10 cm high). After three days of habituation, the animals were submitted to a 10 min acquisition trial (first trial), during which they were placed in the cage in the presence of two identical novel objects (A + A) placed equidistant from each other. A 10 min retention trial (second trial) was performed 2 h later, replacing object A in the cage with object B. Another 10 min retention trial (third trial) was performed 24 h later, replacing object A in the box with object C. The trials were recorded using a camera mounted above the testing box; later, the time that the animal explored the new and the old objects was analyzed. The ratio between the time taken to explore the new object and the time spent on both objects provides an index of recognition memory in the short (2 h) and long term (24 h). In order to avoid object preference biases, the sequence of presentation of the different objects was counter-balanced in each experimental group. The cage and the objects were cleaned with 70° ethanol between different animals to eliminate olfactory cues.

Object location test (OLT). The OLT assesses cognition, specifically spatial memory and discrimination. This test is based on the ability of rodents to recognize when an object has been moved from its previous position and their spontaneous tendency to spend more time exploring the relocated object than the one in a familiar position. Testing was performed in a black box (30 × 40 × 30.5 cm), where the animals are first habituated for 10 min. The next day, two identical objects were introduced in the dark box. Objects were placed equidistant from each other with space around them so that the mice could explore them (A1 + A2). Each mouse was allowed to explore the objects for 5 min. Object exploration was defined as the orientation of the nose to the object at a distance of less than 2 cm. In the second trial conducted 2 h later, the animal again encountered the two objects, but one of them had changed position (A1 + A3). The trials were also video recorded, and for each mouse, the amount of time spent exploring each object was scored. The object location discrimination index was calculated as the ratio between the time spent exploring the relocated object and the total time spent on both objects, in order to evaluate spatial memory. This test was used in the 1-month mCRP exposure study.

Morris water maze test (MWM). The MWM was used to test animals for spatial learning and memory. The test consisted of one day of training and six days of place task learning for spatial reference memory, followed by one probe trial. Mice were trained to locate a hidden platform, 10 cm in diameter, located 20 cm from the wall and 0.5 cm below the water surface. The platform was placed in a circular pool 100 cm in diameter, 40 cm high, with 22–24 °C opaque water, surrounded by black curtains. The animals learned to find the platform using 4 distinctive landmarks as visual cues attached to the pool wall and placed equidistant from each other. The platform was placed between two of these landmarks. Five trial sessions of 60 s per day were performed. In each trial, the mouse was gently released (facing the wall) from one randomly selected starting point (N, S, E or W) and allowed to swim until it escaped onto the platform. Mice that failed to find the platform within 60 s were placed on it for 20 s, the same period as was allowed for the successful animals. On day 7, the platform was removed, and the mice performed a probe trial of 60 s to test learning retention. The computerized tracking system (SMART) was used to measure the distance covered during the learning tasks, along with the time spent in each quadrant of the pool after the removal of the platform in the probe test. The MWM test was used in the 3- and 6-month mCRP exposure studies instead of the OLT test, for a more potent analysis of spatial memory.

### 2.5. Western Blot Analysis of Hippocampus Tissue

After completion of the behavioral tests, mice were sacrificed by dislocation and the brains were dissected on ice to obtain the hippocampus. The tissue was frozen with liquid nitrogen and stored at −80 °C until protein analysis by Western blot, as previously described [41], with some modifications. Hippocampi (right hippocampus samples) were suspended in 15 volumes of ice-cold RIPA buffer (1% IGEPAL, 0.5% sodium deoxycholate, 0.1% SDS in PBS) supplemented with 1 mM orthovanadate, 5 mM sodium fluoride and Complete Protease Inhibitor Cocktail (#11697498001; Roche, Mannheim, Germany). Samples were sonicated and, after centrifuging (13,000× *g*, 10 min, 4 °C), supernatants were collected. The protein concentration of cell lysates was determined using the Bradford protein assay (#5000002; Bio-Rad, Munich, Germany), and equal quantities of proteins (20 μg) were denatured by boiling at 95 °C for 5 min in loading buffer (2% SDS, 10% glycerol, 0.05% bromophenol blue and 50 mM DTT in 50 mM Tris-HCl buffer pH 6.8) and separated by SDS-PAGE at 100 V for 2 h. Polyacrylamide gels were prepared with 30% Acrylamide/Bis Solution 29:1 (#1610156, Bio-Rad) at 10% or 15% according to the target protein size, Tris-HCl/SDS, 10% (*w*/*v*) ammonium persulfate and TEMED [42]. Electrophoresed proteins in the gels were transferred to 0.45 μm PVDF membranes (Immobilon-P, Millipore, Burlington, MA, USA) at 200 mA for 1 h 30 min. The different membranes were blocked for 1 h at room temperature with TBS-T buffer containing 5% Blotting-Grade Blocker (#170-6404; Bio-Rad). Subsequently, the membranes were incubated overnight at 4 °C with primary antibodies diluted 1:1000. Total tau clone HT7 mouse monoclonal antibody (#MN1000; Pierce Endogen, ThermoFisher Scientific, Waltham, MA, USA), p-tau clone AT8 mouse monoclonal antibody (#MN1020; ThermoFisher Scientific), and p-tau Ser396 rabbit polyclonal antibody (#44752G; Life Technologies, Carlsbad, CA, USA) were used for immunodetection of tau-related changes. Mouse monoclonal antibody against amyloid β clone 4G8 (SIG-39220; BioLegend, San Diego, CA, USA) were used to detect increased amyloid pathology. Ionized calcium-binding adapter molecule 1 (Iba1) (#019-19741; Wako; Richmond, VA, Canada) were used to immunodetect activated microglia. Furthermore, monoclonal 8C10 antibody obtained as described above was used to detect mCRP. However, the Western blot technique will not discern between CRP isoforms because the pentameric form will dissociate to monomers in the presence of reducing and denaturing reagents of the blotting buffers [29]. After washing, the corresponding secondary antibody (1:2000) was prepared for 1.5 h incubation at room temperature. Antibodies used for loading control were rabbit polyclonal actin (20–33) (#A5060; Sigma-Aldrich) and monoclonal β-tubulin (#T4026; Sigma-Aldrich) at 1:10,000. Secondary antibodies were peroxidase-conjugated. Antibodies were diluted in the West Vision Block and Diluent SP-7000 (Vector Labs Inc., Burlingame, CA, USA). Proteins were visualized using enhanced chemiluminescence (ECL) detection (Chemidoc™ Imaging System, Bio-Rad, Hercules, CA, USA) and the semi-quantitative fold differences were identified using Image Lab software (v3.0.1; Bio-Rad). Proteins were normalized to actin, to β-tubulin or to the total form for phosphorylated proteins, always analyzed in the same membrane. When p-tau and total tau could not be analyzed in the same membrane, both proteins were previously normalized to actin or to β-tubulin. All membranes contained samples from the control group and the other experimental groups. Normalized densitometry value for each sample was calculated relative to the mean of the values of the control sample in each membrane. To increase the reliability of the results in the mouse Western blots, we analyzed some of the samples in duplicate membranes. In this case, we used the mean value obtained from each hippocampus sample as a value for statistical analysis. Cell culture samples were more readily available, and each well extract was analyzed once.

### 2.6. Quantitative PCR Analysis of Hippocampus Tissue

We determined the transcription levels of the gene Early growth response protein 1 (*Egr1*), as an indicator of hippocampal plasticity underlying neuron dysfunction and memory loss, by real-time quantitative PCR (qPCR). Analysis was performed in the hippocampus of mice submitted to the diverse treatments for 6 months. As a reference for the severity of any decrease found after mCRP treatment, *Egr1* activation was also analyzed in the hippocampus of 5XFAD mice with advanced AD pathology. RNA was extracted from hippocampus samples (left hippocampus) using mirVana miRNA Isolation Kits (#AM156; Life Technologies), following the manufacturer’s instructions, to obtain RNA, including small RNA. The quantity and quality of the RNA samples were determined using a ND-1000 spectrophotometer (NanoDrop Technologies, Wilmington, DE, USA). Random-primed cDNA synthesis was performed using high-capacity cDNA Reverse Transcription Kits (#4368814; Life Technologies). Gene expression of *Egr1* and the reference gene TATA-box binding protein (*Tbp*) was determined using TaqMan Fluorescein amidite (FAM)-labeled specific probes (*Egr1*, #Mm00656724_m1 and Tbp, #Mm00446971_m1; Applied Biosystems) and Quantimix Easy Probe kits (#10.601-4149; Biotools, Madrid, Spain) in an RFX96TM real-time system (Bio-Rad). qPCR assay was run with cDNA obtained from 3.75 ng of RNA. Samples were analyzed in duplicate. Data were normalized to *Tbp* gene expression using the Comparative Cycle Threshold method (ΔΔCT).

The transcription level of the *Crp* gene was also analyzed by qPCR in the hippocampus tissue to discern any contribution of endogenous CRP throughout the 6-month treatment time. For this purpose, we analyzed mouse tissue at 1 month, 3 months and 6 months after CSF or mCRP injection. *Crp* expression was very low in the hippocampus, as expected for brain tissue, and the analysis required a preamplification step. Specific preamplification of *Crp* and *Tbp* cDNA was simultaneously performed using TaqMan Preamp Master Mix (#4391128; Applied Biosystems, Foster City, CA, USA) according to the manufacturer’s instructions. Next, gene expression of *Crp* was determined using TaqMan Fluorescein amidite (FAM)-labeled specific probes (*Crp*, #Mm00432680_g1; Applied Biosystems). qPCR assay was run with cDNA obtained from 10 ng of RNA and submitted to 14 cycles of preamplification. Samples were analyzed in duplicate. Data were normalized to *Tbp* expression after simultaneous preamplification, using the ΔΔC_T_ method. Two samples of liver cDNA from the same strain of mice were used as a positive control.

### 2.7. Assays in the Microglial BV2 Cell Line

We analyzed inflammatory changes in an in vitro experimental setting by the determination of nitric oxide generation in the mouse microglial cell line BV2 (#ATL03001, ICLC, Banca Biologica e Cell Factory, Genova, Italy). BV2 cells were grown in T25 flasks (NuncTM, ThermoFisher Scientific) with culture medium composed of RPMI 1640 with L-glutamine 2 mM, gentamycin 50 µM and 10% heat-inactivated fetal bovine serum (FBS), at 37 °C in a humidified incubator with 5% CO_2_. Cells were sub-cultured at a 1:10 ratio when they reached 80–90% of confluence. Each set of experiments was performed with cells of at least 3 independent passages. For experiments, cells were seeded in 96- or 12-well plates at 2–3 × 10^5^ cells/mL (1.42 × 10^5^ cells/cm^2^). After 24 h, the medium was replaced with fresh culture medium without FBS containing vehicle or anti-inflammatory agents. Anti-inflammatory treatments were TPPU at 50 or 100 µM and 8C10 monoclonal antibody at 1:20 dilution. DMSO 0.1% was used as a vehicle in the TPPU treatment experiments. After 1 h of incubation, the cells were treated with the proinflammatory chemicals lipopolysaccharide (LPS; 0.1 μg/mL) or mCRP (100 µg/mL), and further incubated for 24 h. Native pentameric CRP at 100 µg/mL was also assayed as an additional control.

Nitric oxide generation by activated BV2 was measured by the colorimetric Griess reaction [43] that detects nitrite (NO_2_^−^), a stable reaction product of nitric oxide and molecular oxygen. Briefly, 50 µL of conditioned medium was incubated with 50 µL of Griess reagent for 10 min at room temperature. Optical density was measured at 540 nm using a microplate reader (iEMS Reader MF; Labsystems, Vantaa, Finland). Nitrite concentration was determined from a sodium nitrite standard curve and was then expressed as a percentage of the average maximal values given by the pro-inflammatory agent for each experiment.

Level of inducible nitric oxide synthase (iNOS), the enzyme that catalyzes nitric oxide generation, was analyzed in cell extracts. After incubation, cells were washed with cold PBS and immediately homogenized in ice-cold RIPA buffer supplemented with protease and phosphatase inhibitors. Cell extracts were processed for Western blot analysis following the procedures described for hippocampus tissue extracts. For iNOS immuno-testing, membranes were incubated overnight with purified mouse anti-mouse iNOS clone 54/iNOS (#610431; BD Transduction Laboratories, BD, San José, CA, USA) at the concentration of 1:500.

### 2.8. Statistical Analysis

Results are shown as mean ± SEM. The distribution of the data was checked with the Shapiro–Wilk normality test. Data were analyzed by ANOVA, where not stated otherwise. Results were considered significant when *p* < 0.05. Post-hoc Fisher’s LSD test was performed to compare the means between groups. Statistical analysis was performed using GraphPad Prism v6 (GraphPad Software, San Diego, CA, USA) and IBM SPSS Statistics v23 (IBM Corp., Armonk, NY, USA). Exact N value per group is provided in the figure legends.

## 3. Results

### 3.1. General Indicators of Body Health Were Not Modified by mCRP

Several experimental groups of 3-month-old male C57BL/6J mice were used to analyze the neuroprotection of the antibody 8C10 against mCRP-induced dementia at 1, 3 and 6 months and of TPPU at 1 month, as described in the experimental design (Figure 1a). Body weight did not change significantly in any of the treatment groups in either 1-month study and progressed similarly over time for all the treated groups in the 3- and 6-month studies of mCRP treatment to assay 8C10 protection (Figure 1b) or the 1-month study to assay TPPU protection (Figure 1c) (two-way ANOVA, main effect of age: F (3, 116) = 9.959, *p* < 0.001 and F (6, 203) = 17.79, *p* < 0.001 for 3 and 6 months with 8C10, respectively). The statistical analysis did not show alterations in vertical and horizontal activities or in the pattern of movement evaluated in the open-field test, or in the Sensorimotor tests for motor coordination studies (Supplementary Figure S1). Furthermore, all animals in all experimental groups had preserved vision, as indicated by the presence of visual reflex and posterior leg extension reflex. Body weight, sensorimotor abilities and mobility were recorded as general indicators of the systemic condition of the mice. Visual inspection throughout the study did not reveal any abnormal appearance of the mice or their behavior in the home-cage. Thus, no gross body health alterations were induced by intrahippocampal mCRP treatment or by the neuroprotective treatments with the anti-mCRP antibody 8C10 or the reference anti-inflammatory drug TPPU.

### 3.2. Anti-mCRP Antibody 8C10 Protected Against Anxiety Induced by mCRP at 6 Months of Treatment

**Figure 2:**
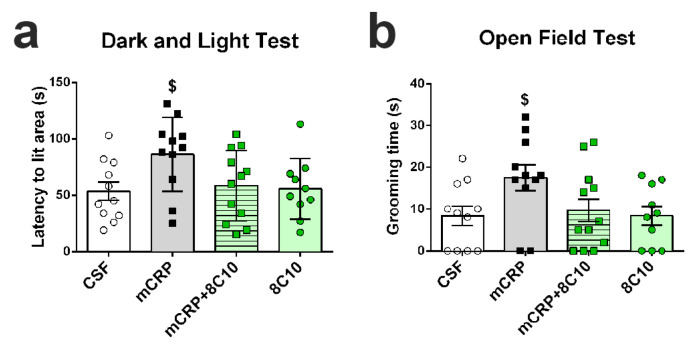
Anxiety induced by mCRP treatment was not evident in the co-treatment with 8C10 antibody. Six months of exposure to mCRP induced traits of anxiety, as shown by increased latency to enter to the lit area in the dark and light test (**a**) and increased grooming time in the open-field test (**b**). 8C10 showed a clear trend toward protecting against anxiety. Values are mean ± SEM (CSF N = 11, mCRP N = 11, mCRP + 8C10 N = 12 and 8C10 N = 10). Statistics: one-way ANOVA showed a trend to significance (**a**) *p* = 0.068, (**b**) *p* = 0.058; ^$^ *p* < 0.05 compared to control by Student’s *t* test.

Tests used to detect BPSD-like behaviors did not show significant effects in the mice treated with mCRP for 1 month or 3 months of exposure. In contrast, mCRP hippocampal injection induced symptoms of anxiety after 6 months of treatment, observed as the trend toward an increased latency to enter the lit area during the dark and light box test (Figure 2a) (one-way ANOVA, F (3, 40) = 2.566, *p* = 0.068; Control vs. mCRP by two-tailed Student’s *t*-test, *t* (20) = 2.381, *p* = 0.027). A similar trend was observed for the increase in grooming time in the open-field test, which is considered an indicator of stress (Figure 2b) (F (3, 40) = 2.701, *p* = 0.058; Control vs. mCRP, *t* (20) = 2.350, *p* = 0.029). These anxiety symptoms at 6 months of treatment were not present with the joint injection of mCRP and the 8C10 antibody, indicating a neuroprotective effect of 8C10. No changes in other BPSD-like behaviors tested, such as neophobia, apathy and depression, were detected in any of the experimental groups, as analyzed in the Corner test, Boissier’s four-hole-board test and Tail suspension test, respectively (Supplementary Figure S2).

### 3.3. Anti-mCRP Antibody 8C10 Protected against Loss of Recognition Memory Induced by mCRP at 1, 3 and 6 Months of Treatment

**Figure 3:**
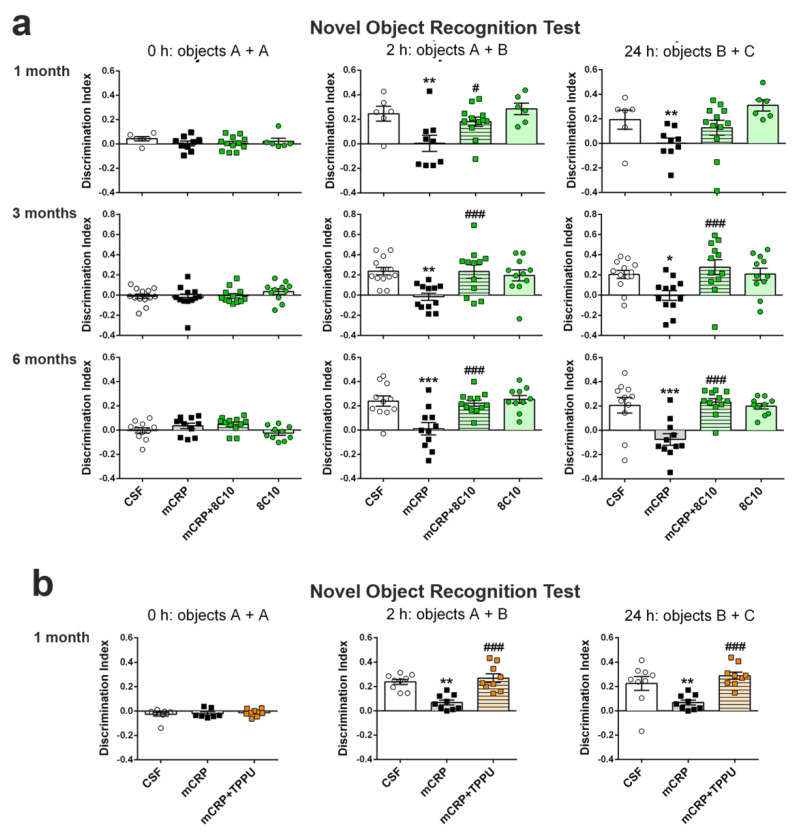
Long-term loss of recognition memory induced by mCRP was prevented by 8C10 antibody. (**a**) 1, 3 and 6 months of exposure to mCRP induced total memory loss shown at 2 and 24 h after an assay of recognition learning in the Novel object recognition test. 8C10 totally prevented memory loss for all the assayed periods. (**b**) TTPU similarly protected against loss of recognition memory after 1 month of exposure to mCRP. Values are mean ± SEM ((**a**) 1 month: CSF N = 6, mCRP N = 9, mCRP + 8C10 N = 12 and 8C10 N = 6; 3 months: CSF N = 13, mCRP N = 12, mCRP + 8C10 N = 12 and 8C10 N = 11; 6 months: CSF N = 11, mCRP N = 11, mCRP + 8C10 N = 12, 8C10 N = 10. (**b**) CSF N = 9, mCRP N = 9 and mCRP + TPP N = 9). Statistics: * *p* < 0.05, ** *p* < 0.01, *** *p* < 0.001, compared to Control group; # *p* < 0.05, ### *p* < 0.001 compared to mCRP group.

The results of the NORT analysis showed a total loss of recognition memory at 1, 3 and 6 months after infusion of mCRP in the mouse hippocampus, but also showed that injection of mCRP with 8C10 monoclonal antibody protected against this loss (Figure 3a) (one-way ANOVA 1-month study, F (3, 29) = 5.081, *p* = 0.006 and F (3, 29) = 4.087, *p* = 0.016, at 2 and 24 h of testing, respectively; 3-month study, F (3, 44) = 6.009, *p* = 0.002, F (3, 44) = 4.680, *p* = 0.006, at 2 and 24 h of testing, respectively; 6-month study, F (3, 40) = 8.755, *p* < 0.001, F (3, 40) = 10.76, *p* < 0.001, at 2 and 24 h of testing, respectively). NORT showed that all mice explored two identical objects for a similar time. However, the mCRP group of mice did not recognize the novel versus the familiar object in the test after a time interval of 2 h or in the retest after a time interval of 24 h. In contrast, the experimental group treated with mCRP plus 8C10 performed the test at the same level as the control group, with similar discrimination indexes (around 0.2 and above), indicating a significantly longer time spent exploring the novel object than the familiar one (see Figure 3a legend for graph details). Therefore, anti-mCRP antibody 8C10 showed neuroprotective effects against mCRP cognitive loss in both short and long time-intervals.

### 3.4. TPPU Protected Against Loss of Recognition Memory Induced by mCRP at 1 Month of Treatment

The reference anti-inflammatory compound TPPU showed a protective effect against the mCRP-induced loss of recognition memory assayed by NORT 1 month after treatment (Figure 3b) (one-way ANOVA, F (2, 24) = 16.48, *p* < 0.0001 and F (2, 24) = 8.613, *p* = 0.0015, at 2 and 24 h of testing, respectively). One animal of the mCRP group was discarded from the basal values because it did not reach a minimum exploration time at 0 h, but showed exploratory activity at 2 and 24 h. Similar to the protective treatment with 8C10, TPPU administered orally to mice injected with mCRP blocked the memory loss that appeared in the mCRP group (see Figure 3b legend for details). Cognitive protection was present at the test time of 2 h and also at the longer test time of 24 h.

### 3.5. Anti-mCRP Antibody 8C10 Protected Against Loss of Spatial Memory Induced by mCRP at 1, 3 and 6 Months of Treatment

**Figure 4:**
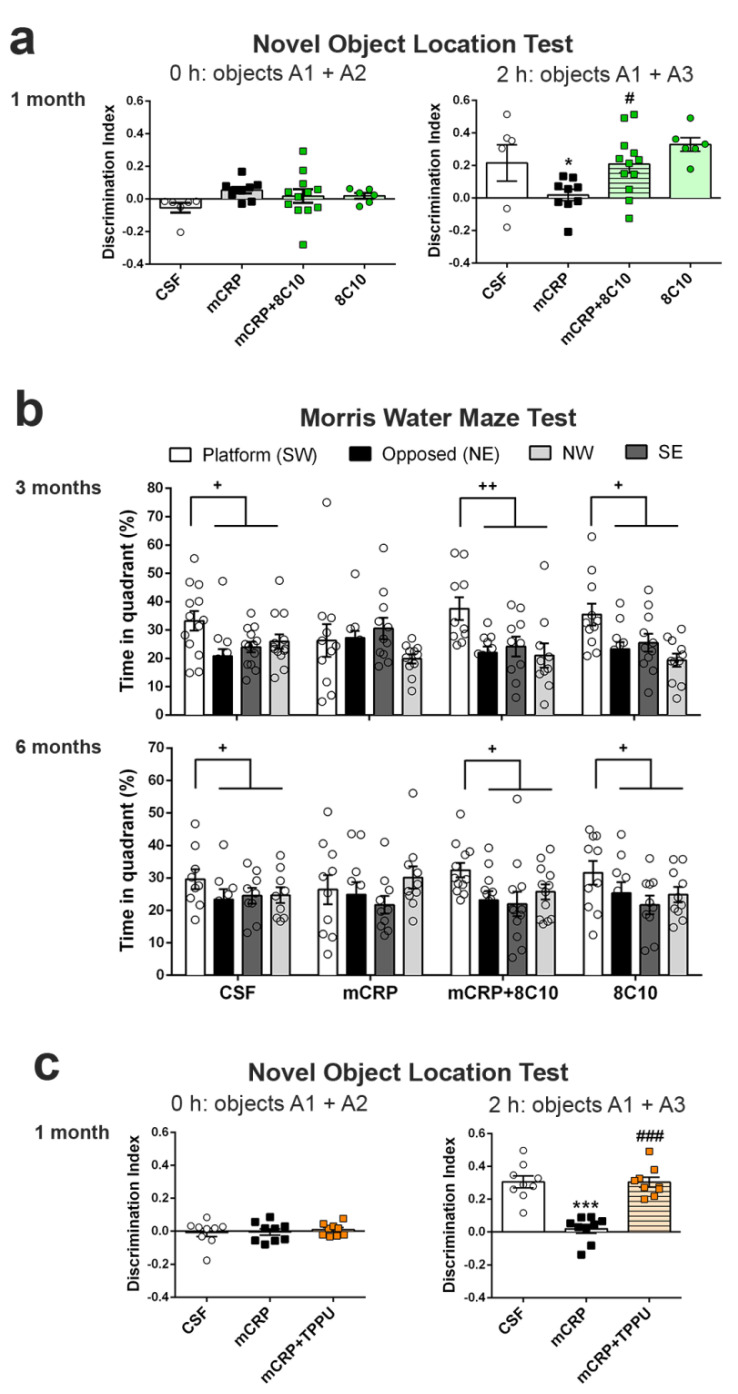
Long-term loss of spatial memory induced by mCRP was prevented by 8C10 antibody. Exposure to mCRP induced total loss of spatial memory, which was prevented by 8C10, as shown by the Novel object location test in the 1-month assessment (**a**) and by the Morris water maze test in the 3 and 6-month assessments (**b**). (**c**) TTPU similarly protected against loss of spatial memory after 1 month of exposure to mCRP, as shown by the Novel object location test. Values are mean ± SEM ((**a**) CSF N = 6, mCRP N = 9, mCRP + 8C10 N = 12 and 8C10 N = 6; (**b**) 3 months, CSF N = 13, mCRP N = 11, mCRP + 8C10 N = 10 and 8C10 N = 11; 6 months, CSF N = 9, mCRP N = 10, mCRP + 8C10 N = 12 and 8C10 N = 10. (**c**) CSF N = 9, mCRP N = 9 and mCRP + TPP N = 9). Statistics: * *p* < 0.05, *** *p* < 0.001, compared to Control group; # *p* < 0.05, ### *p* < 0.001 compared to mCRP group; + *p* < 0.05, ++ *p* < 0.01, Platform quadrant compared to the average of the other quadrants.

The results of the OLT showed a loss of spatial memory response in mCRP-injected mice, but not in the mice also treated with 8C10 antibody (Figure 4a) (one-way ANOVA, F (3, 29) = 4.139, *p* = 0.015 in the study at 1 month). Mice treated with mCRP did not discern between an object that was maintained in the same position and one that had been relocated to a new position at 2 h after previous exploration. In contrast, the experimental group treated with mCRP plus 8C10 performed the test at the level of the control group. These results on spatial memory confirmed the previous protective effects on cognition through recognition memory preservation by 8C10.

For the confirmation of the protective effects of 8C10 after 3 and 6 months of treatment with mCRP, spatial memory was analyzed using the widely known test of MWM instead of OLT. Results of the MWM for the 3- and 6-month studies showed a loss of spatial memory in the mCRP group and total preservation in the mCRP + 8C10 group (Figure 4b) (two-way ANOVA, effect of the percentage of time spent in each quadrant of the pool during the probe test, F (3, 149) = 3.779 *p* = 0.0119 and F (3, 164) = 9.596, *p* < 0.001 for 3- and 6-month treatments, respectively). A few mice that floated instead of actively swimming, as detected in the videotape examination, were not included in the analysis, where discarded animals were: N = 1 in mCRP 3-month group, N = 2 in mCRP + 8C10 3-month group, N = 2 in CSF 6-month group and N = 1 in mCRP 6-month group. Mice in the mCRP groups spent a random amount of time (around 25%) swimming in the pool quadrant where the escape platform was located during the previous acquisition training; however, mice treated with mCRP + 8C10 showed a distinct preference for swimming into the area of the target quadrant, suggesting a preserved spatial memory after 24 h. Otherwise, the acquisition curves, either latency time or swimming distance to the scape platform, did not reach statistical differences between groups, as analyzed by repeated measures ANOVA (Supplementary Figure S3). Therefore, there was some preservation in the learning capacity under our intense protocol of 5 trials per day over 6 days, but memory was totally abolished by mCRP. The group treated with 8C10 alone behaved similarly to the control group treated with vehicle (CSF) in this and all the previous behavioral and cognitive tests.

### 3.6. TPPU Protected against Loss of Spatial Memory Induced by mCRP at 1 Month of Treatment

The protective effect of TPPU against loss of spatial memory induced by mCRP was demonstrated by OLT (Figure 4c) (one-way ANOVA, F (2, 24) = 28.14, *p* < 0.001). Mice injected with mCRP and dosed with TPPU for 1 month were able to recognize the relocated object from the one whose position was maintained 2 h after the previous exploration. Their OLT discrimination index was significantly higher than that of the mCRP-injected mice, which were unable to discriminate between the different spatial positions of the object.

### 3.7. Anti-mCRP Antibody 8C10 Inhibited Tau Hyperphosphorylation Induced by mCRP at 6 Months of Treatment

**Figure 5:**
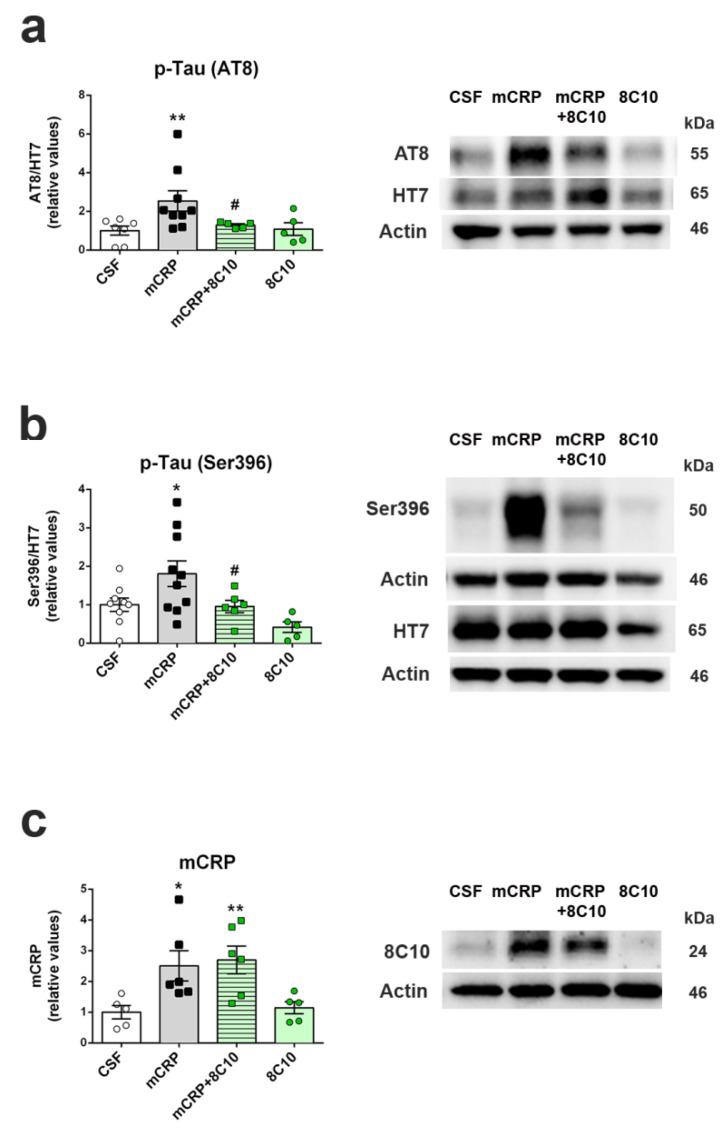
Tau pathology induced by mCRP was prevented by 8C10 antibody. Protein extracts of the whole hippocampus tissue showed higher levels of hyperphosphorylated tau (p-tau) after 6 months of mCRP treatment, but 8C10 prevented this pro-neurodegenerative change. Protein levels detected by p-tau antibodies AT8 (**a**) and Ser396 (**b**) were calculated as a ratio of the levels detected by total tau antibody HT7. (**c**) Protein levels of mCRP detected by 8C10 antibody in the hippocampus 6 months after the intrahippocampal injection were not decreased by co-treatment with 8C10. Representative blots of each experimental group are shown in the same order as the densitometric analysis values in the histograms. The images presented in this figure reproduce the cropped gels, while full-length gels are presented in Supplementary Figures S4, S5 and S8, respectively. Values are mean ± SEM ((**a**) CSF N = 7, mCRP N = 9, mCRP + 8C10 N = 5 and 8C10 N = 5; (**b**) CSF N = 9, mCRP N = 10, mCRP + 8C10 N = 6 and 8C10 N = 5; (**c**) CSF N = 5, mCRP N = 6, mCRP + 8C10 N = 6 and 8C10 N = 6). Statistics: * *p* < 0.05, ** *p* < 0.01 compared to Control group; # *p* < 0.05 compared to mCRP group.

Western blot analysis of tau protein of the hippocampus extracts revealed changes in phosphorylation levels in the 6-month study (Figure 5a,b and Supplementary Figures S4 and S5). Mice treated with mCRP showed significant hyperphosphorylation by two antibodies directed to different tau epitopes that detect pathological tau in AD. Antibodies presented an approximately two-fold increase of p-tau Ser202/Thr305 (clone AT8) (Figure 5a and Supplementary Figure S4) and p-tau Ser396 (Figure 5b and Supplementary Figure S5) by mCRP after normalization for tau content (clone HT7) (one-way ANOVA, F (3, 22) = 3.667, *p* = 0.028 and F (3, 26) = 4.816, *p* = 0.008 for the ratios AT8/HT7 and S396/HT7, respectively). Notably, the hippocampus of mice treated with mCRP and 8C10 showed normalized levels of p-tau as compared to controls. In this group, we discarded an outlier sample that showed very high levels of both AT8 and HT7. Treatment with 8C10 alone did not interfere with the p-tau levels. No changes were detected in the shorter time studies. However, no significant increase of amyloid β in the hippocampus was detected by Western blot after mCRP treatment (Supplementary Figure S6). Therefore, increased p-tau may reflect long-term changes of cognitive loss and neurodegeneration caused by mCRP. Finally, treatment with mCRP or 8C10 antibody did not induce changes in the protein levels of Iba1 (Supplementary Figure S7). Iba1 is a pan-microglial marker, but its expression increases with microglial activation [44]. Therefore, treatments did not cause a generalized activation of microglia to a reactive phenotype, indicating absence of a major inflammatory response.

**Figure 6:**
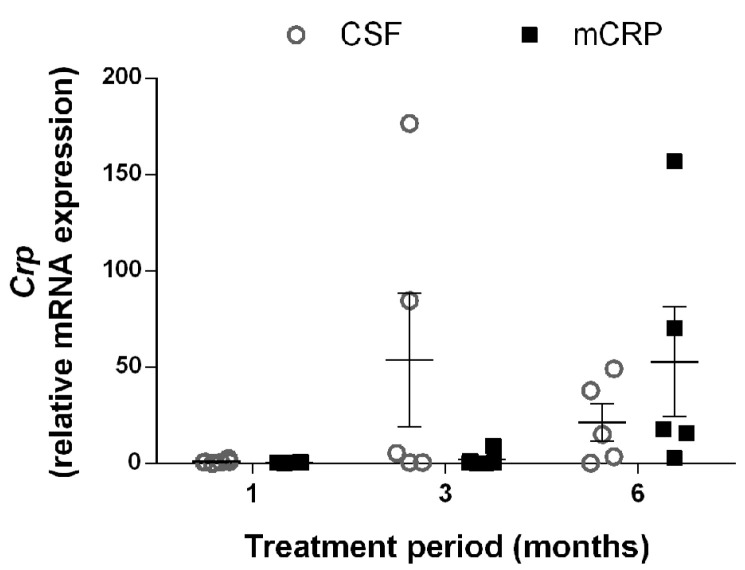
Exposure to mCRP did not induce a significantly increased expression of endogenous *Crp* gene at different times after its injection into the mouse hippocampus. Expression of *Crp* was normalized to the corresponding expression of *Tbp* gene and calculated as fold change of the 1-month CSF group. Values are mean ± SEM (1 month, CSF N = 6, mCRP N = 6; 3 months, CSF N = 5, mCRP N = 6; 6 months, CSF N = 5, mCRP N = 5).

Increased levels of mCRP protein were found in the hippocampus of mice 6 months after the injection (Figure 5c and Supplementary Figure S8) (one-way ANOVA, F (3, 18) = 5.137, *p* = 0.010). Interestingly, mCRP was detected at similar rates in mice receiving treatment with mCRP + 8C10. Proteins are denaturized during the Western blot procedure; therefore, the previous binding of mCRP with 8C10 was destroyed, allowing for fresh 8C10 antibody detection. Pentameric CRP is also disassembled and only mCRP can be detected, as indicated in the Methods Section. However, the 8C10 antibody, although generated against human mCRP, showed immunodetection of mCRP in all the mice and did not discern endogenous from injected mCRP. Nevertheless, transcriptomic analysis showed that *Crp* mRNA levels did not increase significantly in hippocampal tissue after mCRP injection compared to CSF injection (Figure 6) (two-way ANOVA, factors age and treatment, and interaction age x treatment, all *p* > 0.05). Although the specific cDNA preamplification step required for near-undetectable *Crp* expression by standard procedures increased data dispersion, qPCR results ruled out a significant contribution of endogenous CRP or mCRP.

### 3.8. Anti-mCRP Antibody 8C10 Palliated the Decrease of a Neural Plasticity Marker by mCRP at 6 Months of Treatment

**Figure 7:**
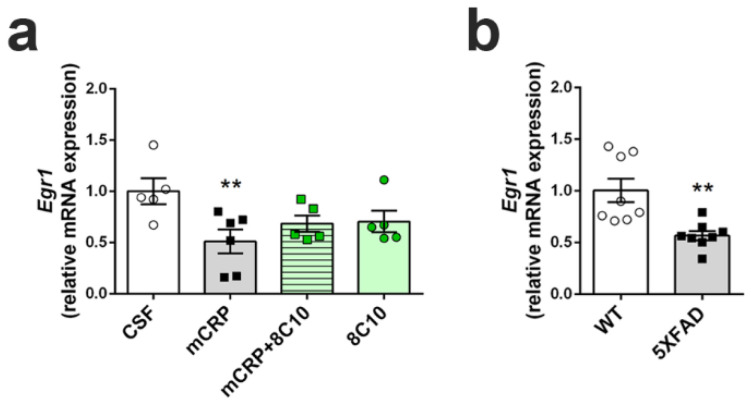
Decreased expression of the neural plasticity marker *Egr1* induced by mCRP was partially prevented by 8C10 antibody. RNA extracts of the whole hippocampus tissue showed reduced levels of expression of the *Egr1* gene after 6 months of mCRP treatment, but 8C10 showed a trend to palliate this decline (**a**). *Egr1* expression was decreased in the hippocampus of 5XFAD mice to a level similar to mCRP-injected mice (**b**). Expression of *Egr1* was normalized to the corresponding expression of *Tbp* gene. Values are mean ± SEM ((**a**) CSF N = 5, mCRP N = 6, mCRP + 8C10 N = 5 and 8C10 N = 5, and (**b**) WT N = 8 and 5XFAD N = 8). Statistics: ** *p* < 0.01 compared to Control group.

The injection of mCRP significantly decreased the levels of *Egr1* mRNA in the hippocampus, as detected at 6 months of treatment, and 8C10 showed a trend to avoid loss of this plasticity marker by maintaining *Egr1* levels in the same range as that of control-treated mice (Figure 7a) (one-way ANOVA, F (3, 17) = 3.525, *p* = 0.037). The protective effect of antibody treatment did not reach statistical significance according to the post hoc analysis (mCRP + 8C10 did not differ from any other group). Therefore, results indicated only a partial prevention of *Egr1* expression loss by 8C10 treatment. In addition, 8C10 may have some unexpected effect that prevented maximum *Egr* expression, as the 8C10 group showed levels similar to the CRP + 8C10 group. Untreated AD transgenic 5XFAD mice showed a similarly reduced level of *Egr1* gene expression as compared to WT mice (Figure 7b) (*t* (14) = 3.590, *p* = 0.0030).

### 3.9. Anti-mCRP Antibody 8C10 and TPPU Reduced Inflammatory Activation of BV2 Cells by mCRP

**Figure 8:**
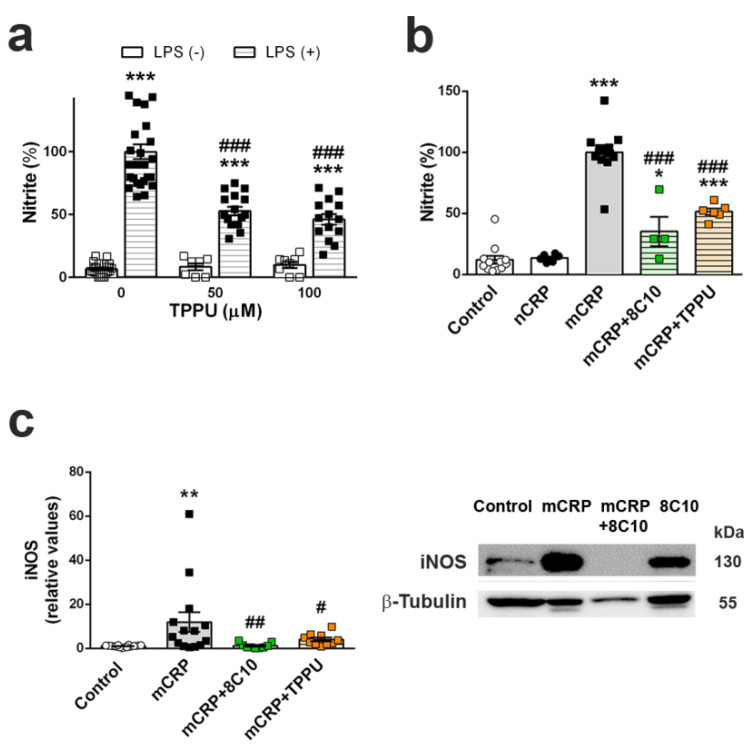
Nitric oxide generation by mCRP in BV2 microglial cells was inhibited by 8C10 and TPPU. (**a**) TPPU decreased the generation of nitric oxide induced by lipopolysaccharide (LPS), as shown by nitrite detection in the culture media of BV2 cells after 24 h of incubation. (**b**) Increased nitric oxide generation by 24 h exposure to mCRP was decreased by both the antibody 8C10 and the anti-inflammatory agent TPPU, while pentameric native CRP (nCRP) had no effect. (**c**) Levels of iNOS protein increased in BV2 cells treated with mCRP, and the increase was inhibited by 8C10 and TPPU. Representative blots of each experimental group are shown in the same order as the densitometric analysis values in the histogram, the blot images reproduce a cropped gel, and full-length gel is presented in Supplementary Figure S10. Values are mean ± SEM ((**a**) TPPU 0 µM Control N = 22, LPS N = 26; TPPU 50 µM Control N = 7, LPS N = 16; TPPU 100 µM Control N = 8, LPS N = 14. (**b**) Control N = 12, CRP N = 6, mCRP N = 12, mCRP + 8C10 N = 4 and mCRP + TPPU N = 6. (**c**) Control N = 18, mCRP N = 14, +TPPU N = 10). Statistics: *** *p* < 0.001 compared to the corresponding Control group, +++ *p* < 0.001 compared to the LPS (+) group without TPPU in (**a**); * *p* < 0.05, *** *p* < 0.001 compared to the Control group, ### *p* < 0.001 compared to mCRP group in (**b**); ** *p* < 0.01 compared to the Control group, # *p* < 0.05 and ## *p* < 0.001 compared to mCRP group in (**c**).

The generation of nitric oxide by BV2 microglial cells was analyzed in order to evaluate the pro-inflammatory effects of mCRP and the protective effects of the 8C10 monoclonal antibody and the anti-inflammatory compound TPPU. First, the anti-inflammatory properties of TPPU in this cell system were demonstrated against a pro-inflammatory stimulus of LPS (Figure 8a) (two-way ANOVA, effect of TPPU F (2, 87) = 18.80, *p* < 0.001, effects of LPS and interaction between the two factors also *p* < 0.001). A significant increase in generation was observed when treating BV2 with LPS 0.1 μg/mL for 24 h in comparison to the basal values in control-treated cells. However, the LPS increase was significantly diminished by TPPU at 50 and 100 μM concentrations.

Next, we evaluated the reaction of microglial BV2 cells to mCRP. In a preliminary assay, we established that the concentration of 100 μg/mL of mCRP showed significant pro-inflammatory effects (Supplementary Figure S9). Nitrite assay demonstrated that mCRP, but not native pentameric CRP, had pro-inflammatory effects on BV2 cells incubated for 24 h, which were protected by both 8C10 (1:20) and TPPU at 100 μM (Figure 8b) (one-way ANOVA, F (4, 35) = 62.62, *p* < 0.001). Therefore, mCRP showed a major pro-inflammatory effect that was inhibited by 8C10 when simultaneously added to the culture medium of BV2 cells. Analysis of iNOS protein levels in the BV2 cell extracts confirmed that mCRP activated the iNOS pathway, and this effect was abolished by 8C10 and partially inhibited by TPPU (Figure 8c and Supplementary Figure S10) (one-way ANOVA, F (3, 47) = 4.555, *p* = 0.004).

## 4. Discussion

Intrahippocampal treatment of mCRP in young adult, 3-month-old mice, induced total memory loss that was detected up to 6 months later when the mice were assessed at 9 months. Here, we extended previous results obtained after 4 weeks of mCRP exposure in this mouse model of dementia [30]. Our results suggest that a single lesion may produce long-lasting cognitive impairment and late anxiety. Mice showed impairment in paradigms for assessing recognition memory and spatial memory at all age points tested. Anxiety behavior shown after 6 months of mCRP exposure is a common BPSD in AD mouse models with advanced pathology [39]. In order to avoid the effects of senescence, we used young animals; however, we may speculate that the dementia induced will be maintained as age advances. Equivalent timing in humans would mean that a single cerebrovascular episode inducing mCRP local deposition [15,30] would cause injuries lasting for a period of more than 20 years [45], and probably for the remainder of the lifetime. The mCRP molecules self-aggregate into diffuse matrix-like structures, reducing aqueous solubility [36]. Indeed, we found significant levels of the injected mCRP remaining inside the hippocampus, and some mCRP molecules were previously detected by immunostaining in blood vessels and neurons of surrounding brain areas in this experimental model [30]. Furthermore, mCRP is mostly associated with human tissue, indicating that it is a tissue-based rather than a serum-based form of this protein [46]. The hippocampus has a central role in learning and memory, and specifically, the targeted CA1 area is a very sensitive region to AD neurodegeneration [47]. Interestingly, long-term potentiation at CA1–CA3 hippocampal synapses is considered the major reflection of synaptic plasticity [48], and we found that injection of mCRP into CA1 reduced both memory responses and gene expression of the transcription factor *Egr1*. *Egr1* regulates many synaptic functions and is specifically involved in long-term potentiation and memory consolidation in the hippocampus [49,50]. It is remarkable that *Egr1* expression decreased to a similar level in mCRP-treated mice as in the 5XFAD transgenic mouse model of AD [38]. 5XFAD mice suffer from early memory loss [51], as we showed here for mCRP mice. Overall, then, these factors validate our model of mCRP-induced dementia. Considering mCRP as a significant contributor to the triggering of AD after stroke [14,30] and to the progression of neuroinflammation and loss of synaptic function in AD [14], effective treatments against it may be able to decrease poststroke AD cases and other diseases involving CRP activation.

The antibody against mCRP, 8C10, protected against the memory loss induced by mCRP at the three treatment periods assayed up to a maximum of 6 months of exposure. Both spatial memory and recognition memory were preserved by 8C10 in mice, which showed the same level of test performance as control mice despite mCRP dosing. However, we did not find significant protection against the decrease in the *Egr1* neuroplasticity marker by 8C10. Although an upward trend in *Egr1* may have caused some improvements in neuroplasticity, other pathways may be involved, and future studies to discern specific neuroprotective mechanisms are warranted. A trend toward higher anxiety in mCRP mice was also prevented by 8C10, indicating protection against the spread of damage from the hippocampus to connected brain areas. Indeed, the mCRP protein was detected in the hippocampus of the mice injected with mCRP, but also in those injected with mCRP and the 8C10 antibody. Therefore, it is likely that the antibody blocks the active region of the mCRP molecule and that this is enough to protect against any damage inflicted to the hippocampal tissue. It is known that 8C10 binds to the N-terminal part of human mCRP through aa 22–45, and that this may not allow mCRP binding to cholesterol through aa 35–47, thus preventing mCRP anchorage to the lipid rafts microdomains, which is considered its mechanism of cell interaction [13,52]. Protection by 8C10 was maintained throughout the 6-month period, and therefore it is not anticipated that mild aggregation changes of deposited mCRP molecules [36] may diminish their affinity for the antibody.

Tau pathology in the hippocampus of mCRP-injected mice was avoided with 8C10. Increases in immunostaining of p-tau have been found in neurons of brain tissue one month after mCRP intrahippocampal injection, and a double immunofluorescence analysis has demonstrated neuronal co-localization of mCRP and p-tau [30]. Here, the high levels of p-tau reached at several pathological sites after 6 months of mCRP damage were readily detected by Western blot. The high levels of p-tau were paralleled by mCRP deposits also detected by Western blot. These results are supported by previous histological demonstration of mCRP in the brain one month after its experimental injection [30,32]. Furthermore, we can speculate on an exogenous origin of the mCRP deposits according to the lack of increased gene expression of *Crp* at any of the analyzed time points of 1 month, 3 months and 6 months after surgery. If this were the case, the long-term neuroprotection attained with 8C10 despite the maintained presence of exogenous mCRP into the mouse brain is intriguing and warrants further analysis of the mCRP-antibody dynamics. Interestingly, in vitro experiments with cultured neurons and bovine aortic endothelial cells incubated with mCRP have also shown increased p-tau levels, which may be protected by 8C10 [30]. Therefore, our overall in vivo and previous in vitro results suggest that the prevention of mCRP interaction with the cell membrane by the antibody avoids the subsequent activation of cellular cascade systems, leading to a neurodegeneration stage reflected by tau pathology and loss of synaptic function. Tau pathology is a hallmark of AD and other dementia types and its progression correlates with AD stages and dementia severity [53]. It is also involved in the chain of poststroke neurodegenerative changes leading to dementia; specifically, increased p-tau was found in tissue from infarcted patients [14] and also in experimental models of cerebral hypoperfusion [54] and ischemia [55]. Protection of tau pathology by 8C10 in vivo confirms the potential of the antibody against the potential development of neurodegeneration by mCRP. Increase of pathological tau phosphorylation by mCRP is in agreement with the activation of the p38 MAPK pathway as a cytotoxicity effector of mCRP, that has been identified in human coronary artery endothelial cells [56,57,58] and human U937 macrophages [13]. Activation of p38 MAPK is involved in tau phosphorylation and in the pathological changes in AD [59], and would support tau pathology and AD traits of the mCRP mouse model.

Microglia cells are the first-line immune defense in the brain and are key players in neuroinflammatory processes. Activated microglia increase their phagocytic activity and release oxygen species and inflammatory molecules, as do macrophages. The direct activation of microglia by mCRP has not been demonstrated, although mCRP can be detected in microglia cells alongside neurons in areas surrounding the peri-infarcted regions of ischemic stroke tissue [30]. The absence of significant microgliosis in the mCRP mouse model, as indicated by unaltered Iba1 levels in the whole hippocampus extracts, may be due to a local effect on microglia cells next to mCRP deposition sites, and this would need confirmation in future studies. Here, we demonstrated that mCRP, but not CRP, activated the mouse microglial cell line BV2, as shown by the increased generation of nitric oxide. Notably, 8C10 blocked this effect, therefore preventing the activation of the inducible nitric oxide synthase (iNOS) pathway by mCRP. mCRP has also been shown to increase iNOS levels and NO generation in the human macrophage-like cell line U937 [60]. Overactivated iNOS has been implicated in many inflammatory pathologies, including AD [61]. It is plausible that a vicious or sustained activation of microglia by mCRP may induce a spiral of neuroinflammation and neurodegeneration [62]. In addition, microglia perform a wide range of physiological functions in normal conditions, contributing to brain homeostasis and neuron functionality [63]; therefore, we may speculate that microglia activation by mCRP makes a key contribution to the poststroke dementia and AD.

The pro-inflammatory mechanisms of mCRP are not fully known, but it is known to cause the activation of platelets, leukocytes and endothelial cells, and also of complement via C1q binding [12], all of which may potentially lead to a cascade of inflammatory damage in the cerebral microvasculature. For instance, high levels of mCRP have been detected in the AD microvasculature positive for the neovascularization marker CD105, suggesting activation and possible potential for aberrant angiogenesis [30]. Furthermore, the blood–brain barrier may be damaged in infarcted areas, facilitating the infiltration of activated leukocytes and mCRP and thus promoting poststroke AD. Finally, a sustained interaction between mCRP-induced brain pathology and AD pathology itself would further activate neuroinflammation and associated neurodegeneration [10]. For instance, mCRP is also generated by CRP interaction with amyloid β plaques in AD brain [15].

TPPU-induced neuroprotection against memory loss in mCRP-injected animals confirms the presence of neuroinflammation and related neurodegenerative processes underlying this mouse model of dementia. TPPU is a well-characterized soluble epoxide-hydrolase inhibitor that can readily cross the blood–brain barrier. Inhibition of this enzyme in order to halt the hydrolysis of beneficial epoxyeicosatrienoic acids to their corresponding metabolites is a novel approach against neuroinflammatory pathologies [35]. TPPU has been found to be neuroprotective in experimental models of chronic hypoperfusion [64], reperfusion after focal ischemia [65] and AD [37]. Soluble epoxide hydrolase blockade by another experimental drug has proven to reduce microglia activation in vivo, induced by experimental traumatic brain injury, and in vitro, proven in BV2 cells injured with LPS [66]. In line with these findings, we showed that TPPU is protective against the increase of nitric oxide by LPS in BV2 microglial cells. We also demonstrated that TPPU reduced the activation of the pro-inflammatory pathway of nitric oxide activation induced by mCRP incubation. Finally, we showed a differential effect of mCRP and native CRP in the iNOS activation, suggesting that this pathway is a target effector of mCRP neuroinflammation.

## 5. Conclusions and Future Directions

In conclusion, in this study, we provided proof of the long-term deleterious effects of mCRP after its deposition in the hippocampus of mice. The induction of processes promoting neurodegeneration as shown by increased p-tau in vivo and nitric oxide in BV2 microglial cells can be blocked by the 8C10 antibody, which is specific for the monomeric CRP but does not associate with the pentameric CRP. Furthermore, using TPPU confirmed that the proinflammatory pathways are the main inducers of neuronal damage by mCRP. The induction of dementia with AD traits by mCRP confirms the link between cerebrovascular injury and AD and identifies mCRP as a druggable therapeutic target.

Specific blockade of mCRP is a promising therapy for reducing the neurodegeneration after a cerebrovascular injury and the development of AD, the most common form of dementia in the elderly population. Furthermore, mCRP can be a useful biomarker of the prognosis of neurodegenerative processes associated with neuroinflammation, when it could be detected by non-invasive techniques. Advances in mCRP detection and therapeutic approaches open up new avenues in the fight against all the pathological conditions affected by the damaging interaction between mCRP and cells and tissues.

## 6. Limitations

The simultaneous injection of 8C10 antibody with mCRP demonstrated the beneficial effects of mCRP blockade that happen most probably before it can enter into the cells, but cannot be used as an antibody therapy test. Subsequent in vivo assays for immunotherapy require independent administration of the neutralizing monoclonal antibody 8C10 at different times after mCRP injury, using a peripheral dosing regimen tailored to its hitherto unknown bioavailability and pharmacokinetic properties. Furthermore, intracellular delivery of the antibody to directly block mCRP in the cytoplasm of neurons will require the development of a specific carrier system across cell membranes [67].

Additional control groups were not considered at this point, so as to use the minimum number of animals. However, the inclusion of control antibody groups with a scrambled 8C10 protein injected together with CSF and with mCRP, or the assay of other potential candidate antibodies, would be required to fully characterize the effects of mCRP blockade. Furthermore, a control TPPU group would be desirable, although this agent is known not to cause harmful effects in mice at the dose used [37].

Here, we did not trace the injected mCRP throughout the timeline. Assessment of the exact localization and stability of mCRP deposits in both mCRP- and mCRP + 8C10-injected hippocampus and the weight of partial spreading to other brain areas [32] would be convenient for a comprehensive characterization of the model of mCRP dementia in mice.

## Data Availability

Data supporting the conclusions of this article are contained in the corresponding figures and supplementary materials; full raw data will be made available by the authors without undue reservation.

## Supplementary Materials

The following are available online at https://www.mdpi.com/article/10.3390/biomedicines9070828/s1, Figure S1: Sensorimotor ability and patterns of mobility after 6 months of mCRP and 8C10 treatments, Figure S2: Behaviors of neophobia, depression and apathy after 6 months of mCRP and 8C10 treatments, Figure S3: Acquisition of learning in the Morris Water Maze Test after 3 and 6 months of mCRP and 8C10 treatments, Figure S4: Blots of the cropped image shown in Figure 5a, Figure S5: Blots of the cropped image shown in Figure 5b, Figure S6: Protein levels of amyloid β in the hippocampus tissue after 6 months of mCRP and 8C10 treatments, Figure S7: Protein levels of Iba1 in the hippocampus tissue after 6 months of mCRP and 8C10 treatments, Figure S8: Blots of the cropped image shown in Figure 5c, Figure S9: Nitric oxide generation by mCRP in BV2 microglial cells, Figure S10: Blots of the cropped image shown in Figure 7c.
