# Supplementary material for: Antibody Protection against Long-Term Memory Loss Induced by Monomeric C-Reactive Protein in a Mouse Model of Dementia
Source: Biomedicines. 2021 Jul 16;9(7):828. doi: 10.3390/biomedicines9070828 (PMC8301488; doi:10.3390/biomedicines9070828)
Supplement: Supplementary file 1 [file biomedicines-09-00828-s001.zip › biomedicines-1231549-SI.pdf]

Supporting information

Antibody protection against long-term memory loss induced by monomeric C-reactive protein in a mouse model of dementia

Elisa García-Lara, Samuel Aguirre, Núria Clotet, Xenia Sawkulycz, Clara Bartra, Lidia Almenara, Cristina Suñol, Rubén Corpas, Montserrat Carrascal, Peter Olah, Florin Tripon, Andrei Crauciuc, Mark Slevin, Coral Sanfeliu

E-mail corresponding author: coral.sanfeliu@iibb.csic.es

Supplementary Figure S1. Sensorimotor ability and patterns of mobility after 6 months of of mCRP and 8C10 treatments.

Supplementary Figure S2. Behaviors of neophobia, depression and apathy after 6 month of mCRP and 8C10 treatments.

Supplementary Figure S3. Acquisition of learning in the Morris Water Maze Test after 3 and after 6 month of mCRP and 8C10 treatments.

Supplementary Figure S4. Blots of the cropped image shown in Figure 5a.

Supplementary Figure S5. Blots of the cropped image shown in Figure 5b.

Supplementary Figure S6. Protein levels of amyloid β in the hippocampus tissue after 6 month of mCRP and 8C10 treatments.

Supplementary Figure S7. Protein levels of Iba1 in the hippocampus tissue after 6 month of mCRP and 8C10 treatments.

Supplementary Figure S8. Blots of the cropped image shown in Figure 5c.

Supplementary Figure S9. Nitric oxide generation by mCRP in BV2 microglial cells.

Supplementary Figure S10: Blots of the cropped image shown in Figure 7c.

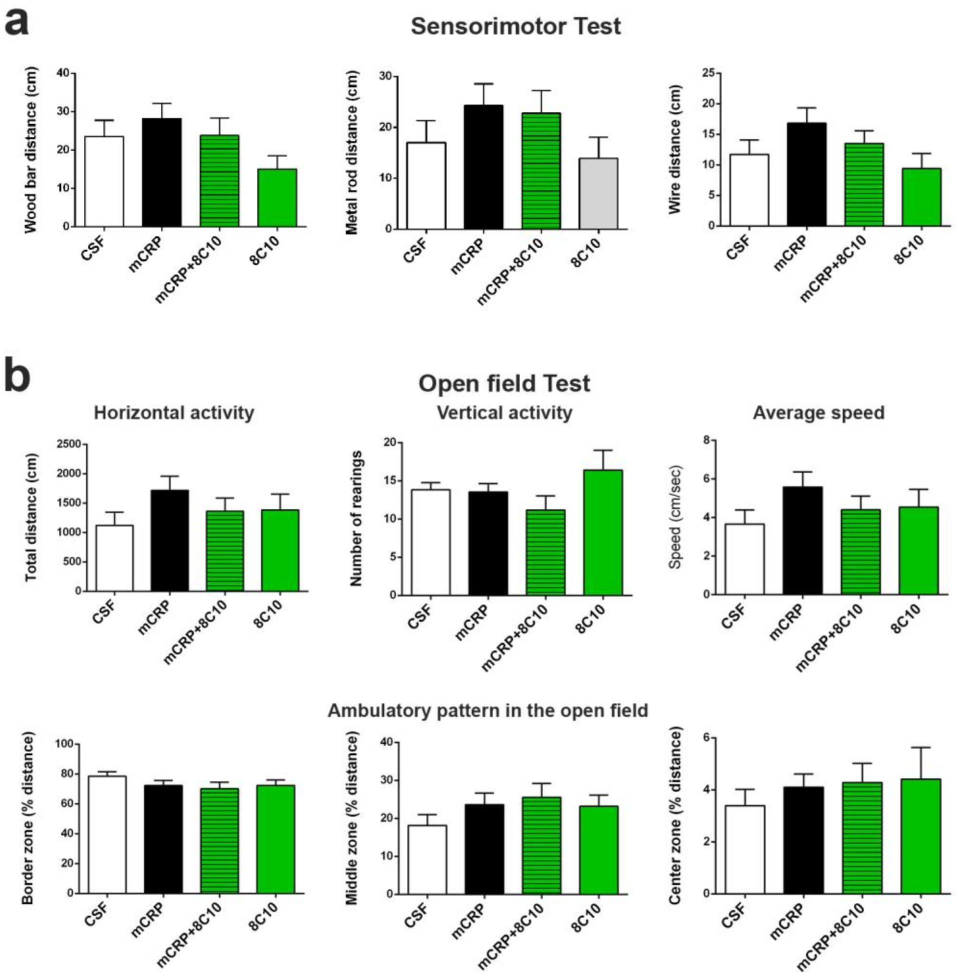

Supplementary Figure S1. Sensorimotor ability and patterns of mobility after 6 months of of mCRP and 8C10 treatments. (a) No effects were detected in the distance covered in the wood bar, metal rod or wire hang test, as indicated. **(b)** No effects were detected in the total activities, average speed or the ambulatory pattern in the Open field test.

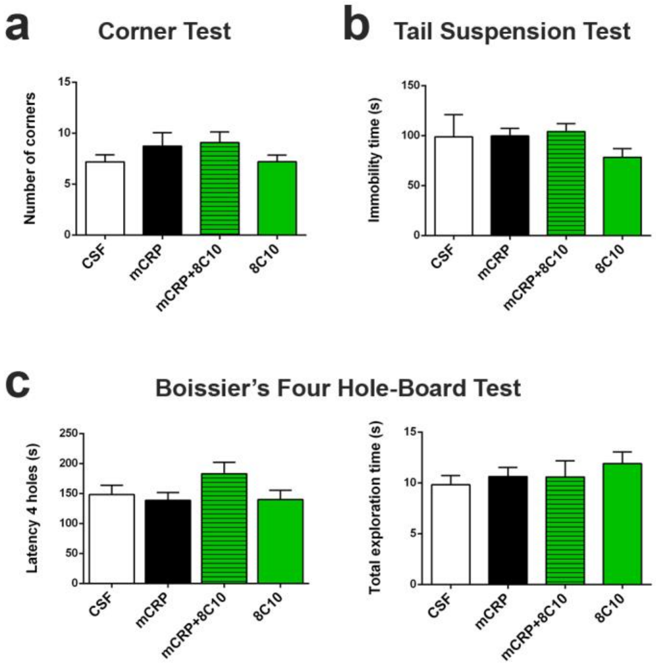

Supplementary Figure S2. Behaviors of neophobia, depression and apathy after 6 month of mCRP and 8C10 treatments. No effects were detected in neophobia, depression and apathy, as measured by the Corner test **(a)**, the Tail suspension test **(b)** and the Boissier's four hole-board test **(c)**, respectively.

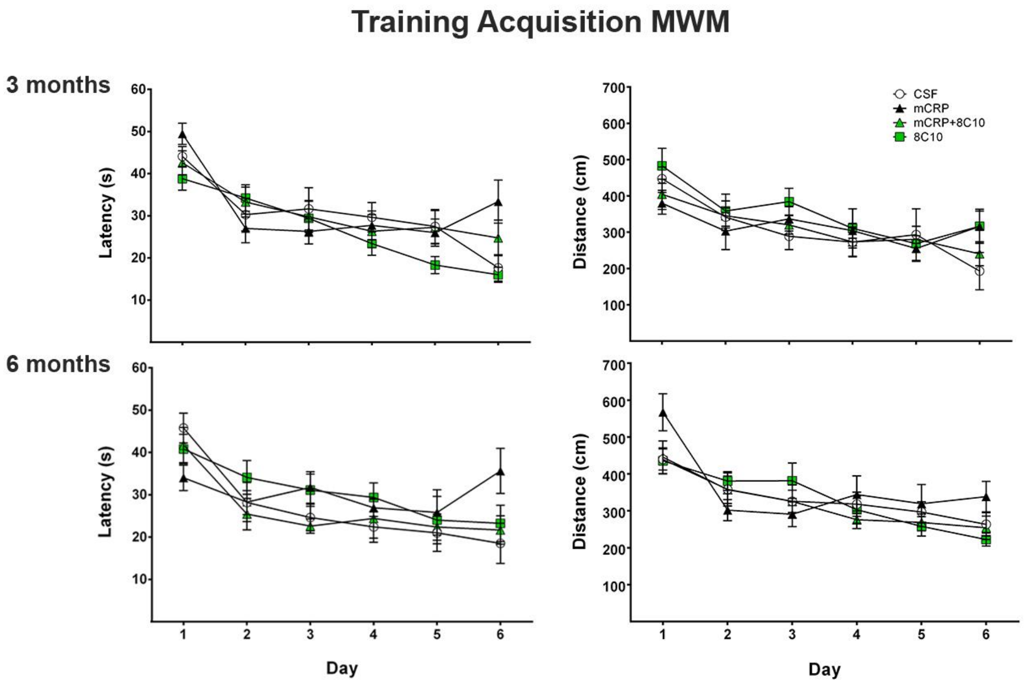

Supplementary Figure S3. Acquisition of learning in the Morris Water Maze (MWM) Test after 3 and after 6 month of mCRP and 8C10 treatments. There were no statistically significant differences between the treatment groups in the latency time or the swimming distance to reach the scape platform in the successive days of training. Repeated measures ANOVA showed an effect of the day ($P < 0.001$) for all parameters.

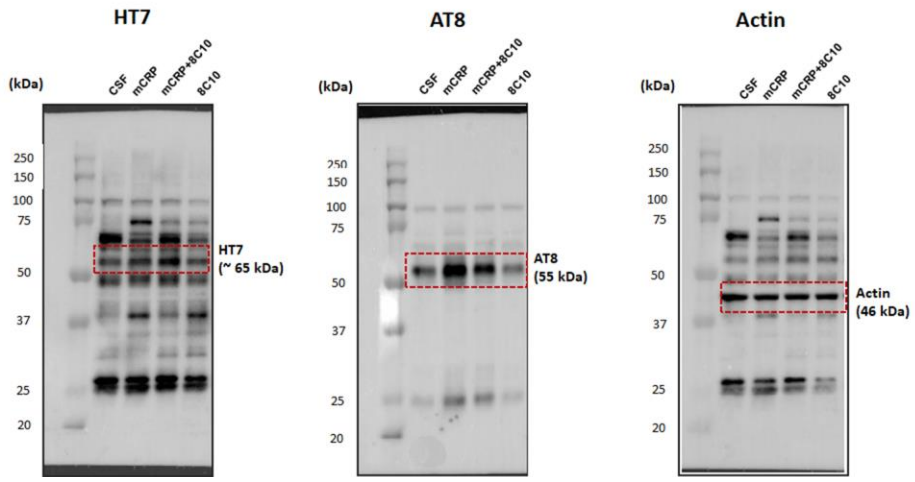

Supplementary Figure S4. Blots of the cropped image shown in Figure 5a. The cropped areas are labeled with red-dotted boxes. These images represent the full length of the same gel (10% acrylamide) Loading control was included in the left lane.

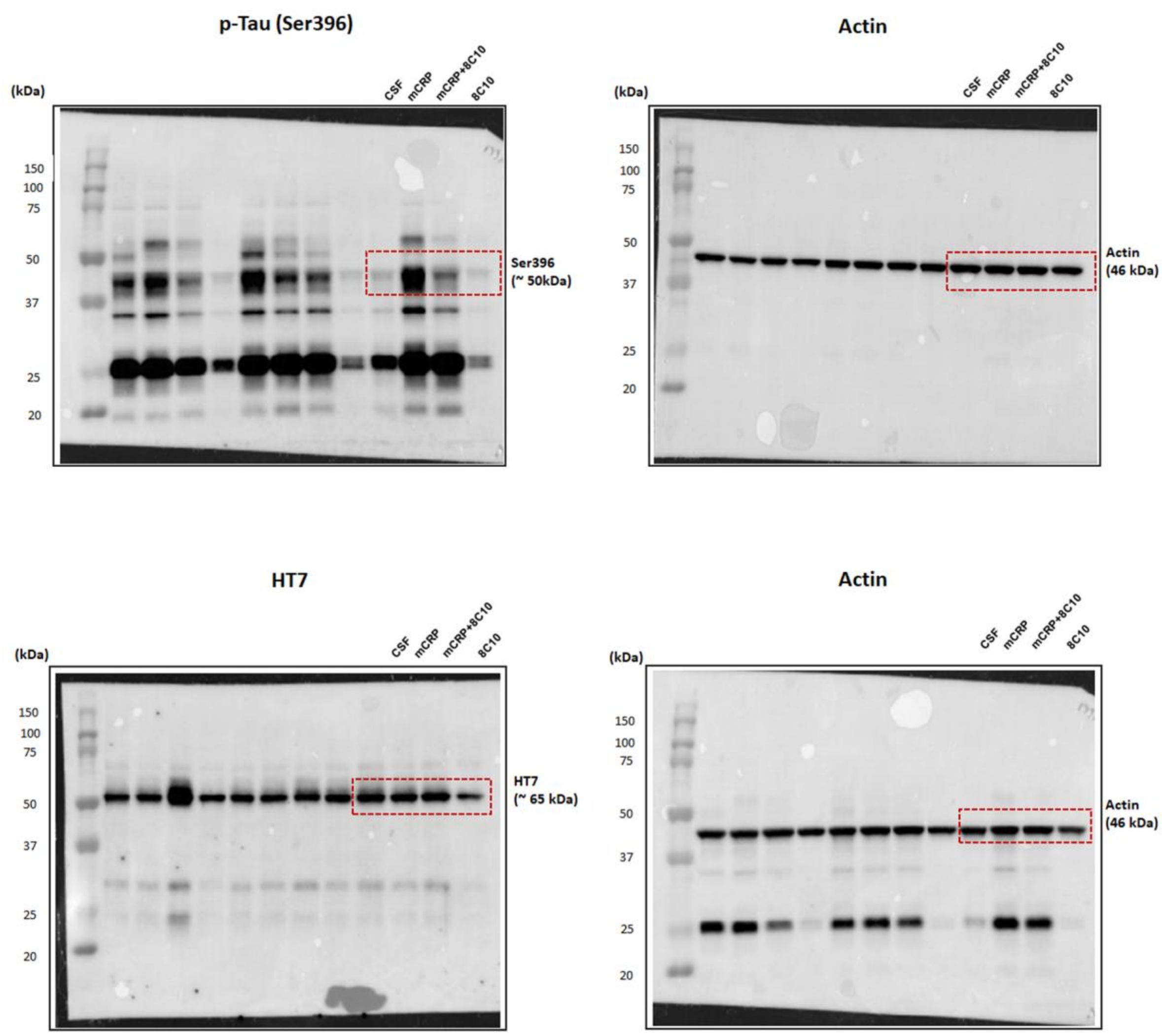

Supplementary Figure S5. Blots of the cropped image shown in Figure 5b. The cropped areas are labeled with red-dotted boxes. These images represent the full length of two different gels (10% acrylamide): top, p-Tau (Ser396) and Actin and bottom, HT7 and Actin. Loading control was included in the left lane of both gels.

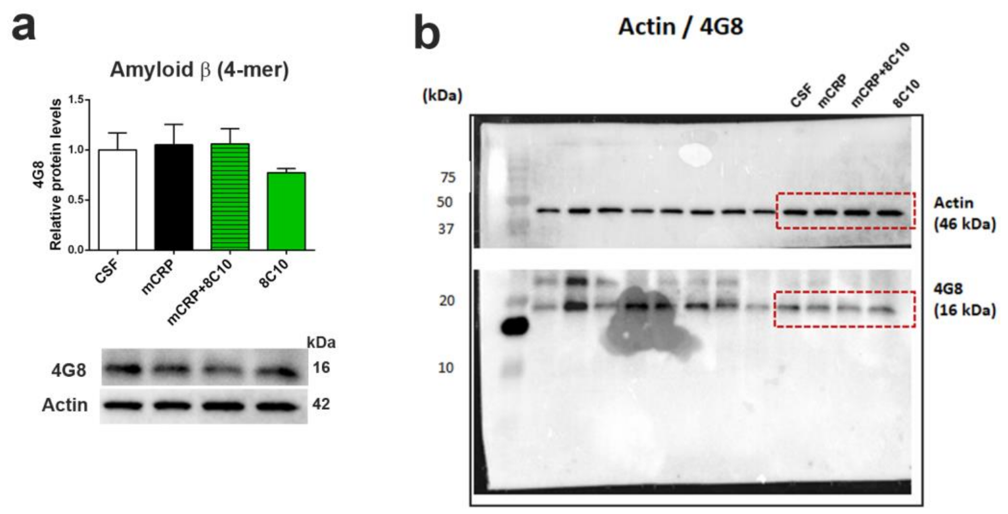

Supplementary Figure S6. Protein levels of amyloid β in the hippocampus tissue after 6 month of mCRP and 8C10 treatments. No significant changes of amyloid β were detected in the hippocampus tissue by Western blot**. (a)** Representative blots of each experimental group are shown in the same order as the densitometric analysis values in the histograms. **(b)** Full length gel blots. These images represent the grouping of the same gel (15% acrylamide) cropped by the middle. Loading control was included in the left lane.

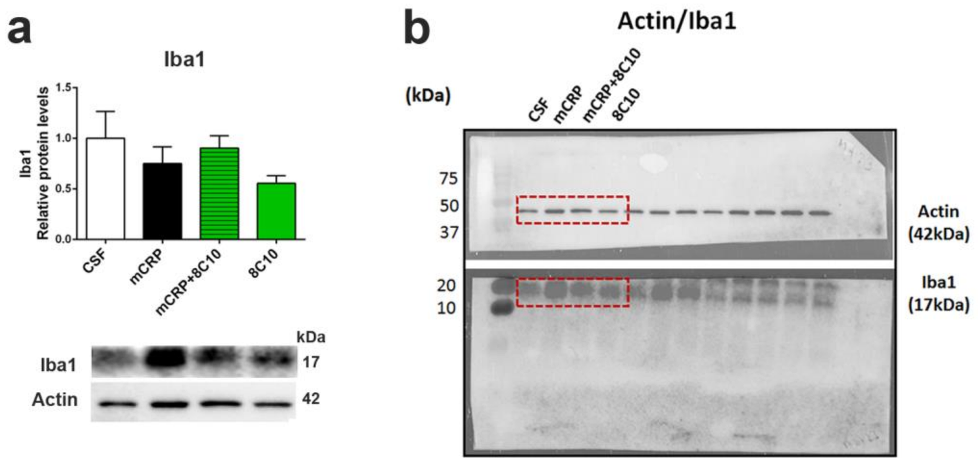

Supplementary Figure S7. Protein levels of Iba1 in the hippocampus tissue after 6 month of mCRP and 8C10 treatments. No significant changes of Iba1 were detected in the hippocampus tissue by Western blot**. (a)** Representative blots of each experimental group are shown in the same order as the densitometric analysis values in the histograms. **(b)** Full length gel blots. These images represent the grouping of the same gel (15% acrylamide) cropped by the middle. Loading control was included in the left lane.

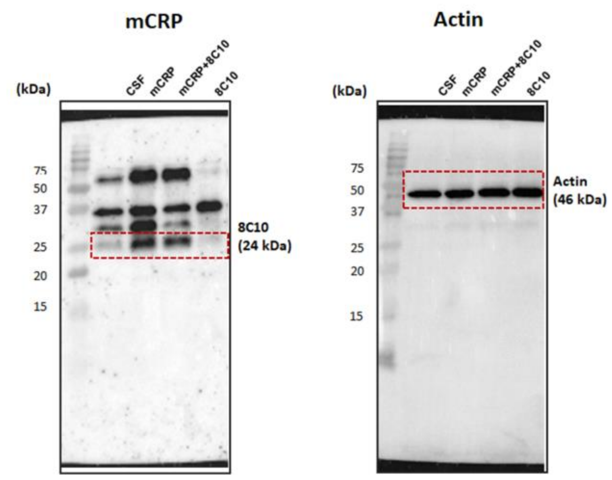

Supplementary Figure S8. Blots of the cropped image shown in Figure 5c. The cropped areas are labeled with red-dotted boxes. These images represent the full length of the same gel (15% acylamide). Loading control was included in the left lane.

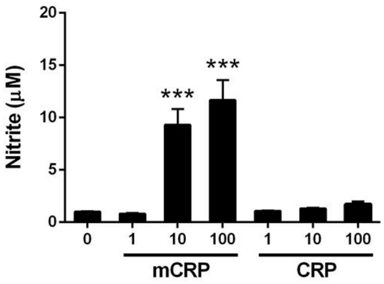

Supplementary Figure S9. Nitric oxide generation by mCRP in BV2 microglial cells. mCRP, but not native pentameric CRP, increased the generation of nitric oxide as detected by nitrite determination in the culture medium.

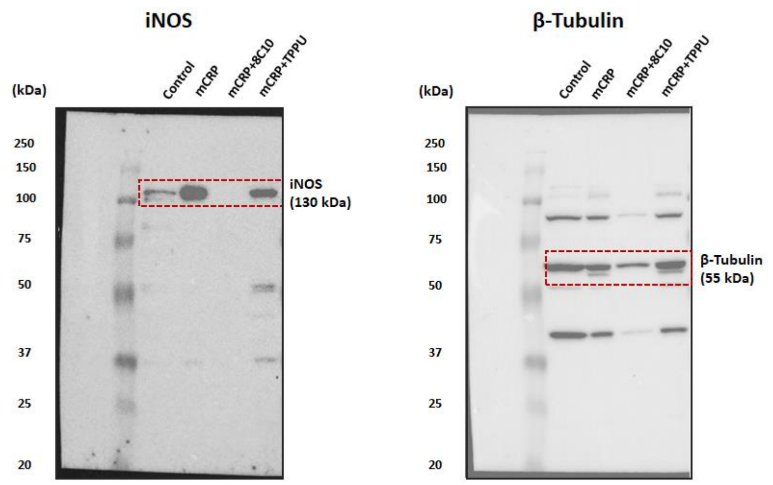

Supplementary Figure S10. Blots of the cropped image shown in Figure 7c. The cropped areas are labeled with red-dotted boxes. These images represent the full length of the same gel (10% acylamide). Loading control was included in the left lane.
